# Structure and Magnetic Properties of Thermodynamically Predicted Rapidly Quenched Fe_85-x_Cu_x_B_15_ Alloys

**DOI:** 10.3390/ma14247807

**Published:** 2021-12-16

**Authors:** Lukasz Hawelek, Tymon Warski, Adrian Radon, Adam Pilsniak, Wojciech Maziarz, Maciej Szlezynger, Mariola Kadziolka-Gawel, Aleksandra Kolano-Burian

**Affiliations:** 1 Lukasiewicz Research Network-Institute of Non-Ferrous Metals, 5 Sowinskiego St., 44-121 Gliwice, Poland; tymon.warski@imn.lukasiewicz.gov.pl (T.W.); adrian.radon@imn.lukasiewicz.gov.pl (A.R.); adam.pilsniak@imn.lukasiewicz.gov.pl (A.P.); aleksandra.kolano-burian@imn.lukasiewicz.gov.pl (A.K.-B.); 2 PhD School, Faculty of Mechanical Engineering, Silesian University of Technology, Konarskiego 2a St., 44-100 Gliwice, Poland; 3 Department of Measurement Science Electronics and Control, Silesian University of Technology, 10 Akademicka St., 44-100 Gliwice, Poland; 4 Institute of Metallurgy and Materials Science Polish Academy of Sciences, 25 Reymonta St., 30-059 Krakow, Poland; w.maziarz@imim.pl (W.M.); m.szlezynger@imim.pl (M.S.); 5 A. Chelkowski Institute of Physics, University of Silesia, 1 75 Pulku Piechoty St., 41-500 Chorzow, Poland; mariola.kadziolka-gawel@us.edu.pl

**Keywords:** soft magnetic materials, materials characterization, toroidal cores, crystal structure, magnetic properties

## Abstract

In this work, based on the thermodynamic prediction, the comprehensive studies of the influence of Cu for Fe substitution on the crystal structure and magnetic properties of the rapidly quenched Fe_85_B_15_ alloy in the ribbon form are performed. Using thermodynamic calculations, the parabolic shape dependence of the Δ*G^amoprh^* with a minimum value at 0.6% of Cu was predicted. The Δ*G^amoprh^* from the Cu content dependence shape is also asymmetric, and, for Cu = 0% and Cu = 1.5%, the same Δ*G^amoprh^* value is observed. The heat treatment optimization process of all alloys showed that the least lossy (with a minimum value of core power losses) is the nanocomposite state of nanocrystals immersed in an amorphous matrix obtained by annealing in the temperature range of 300–330 °C for 20 min. The minimum value of core power losses P_10/50_ (core power losses at 1T@50Hz) of optimally annealed Fe_85-x_Cu_x_B_15_ x = 0,0.6,1.2% alloys come from completely different crystallization states of nanocomposite materials, but it strongly correlates with Cu content and, thus, a number of nucleation sites. The TEM observations showed that, for the Cu-free alloy, the least lossy crystal structure is related to 2–3 nm short-ordered clusters; for the Cu = 0.6% alloy, only the limited value of several α-Fe nanograins are found, while for the Cu-rich alloy with Cu = 1.2%, the average diameter of nanograins is about 26 nm, and they are randomly distributed in the amorphous matrix. The only high number of nucleation sites in the Cu = 1.2% alloy allows for a sufficient level of grains’ coarsening of the α-Fe phase that strongly enhances the ferromagnetic exchange between the α-Fe nanocrystals, which is clearly seen with the increasing value of saturation induction up to 1.7T. The air-annealing process tested on studied alloys for optimal annealing conditions proves the possibility of its use for this type of material.

## 1. Introduction

Nanocrystalline and amorphous Fe-based soft magnetic materials have been known for many years [1,2,3]. They are still promising for power electronics applications. The magnetic properties of soft magnetic materials depend on the chemical composition and proper annealing process [4]. The high saturation induction nanocrystalline alloy systems including FeBCu [5,6,7], FeSiBCu [8], and FeSiBPCu [9] were successfully developed over the last two decades. For all of these alloys, the maximum values of magnetic saturation were obtained for Cu content chemical compositions using the conventional heating rate of 10–40 °C/min and subsequent isothermal annealing. As it was shown by T. Liu et al. [10] for the conventional annealing, the appropriate addition of Cu synchronizes the process of crystallization, leading to the uniform microstructures with fine grains formation. This is essential for excellent magnetic properties. They also showed that for these alloys, the microstructure evolves during the annealing process via three stages: the stress relief stage, nanocrystallization stage, and second phase precipitation stage.

Lastly, starting from the binary Fe_86_B_14_ alloy by use of the thermodynamic calculation approach, comprehensive thermal analysis, crystal structure, and magnetic studies, a full conventional annealing optimization of Fe_86-x_Cu_x_B_14_ alloys was presented [6]. From the investigations of the thermodynamic parameters, it was found that for the x = 0.55 alloy, the Gibbs free energy of amorphous phase formation has a global minimum value. Calculated from the thermal analysis, the average activation energy of α-Fe phase crystallization also showed a minimum value similar to the Cu-free Fe_86_B_14_ alloy. From the annealing optimization process, optimal soft magnetic properties were obtained for the nanocrystalline structure for the annealed sample in the temperature range 290–320 °C for 20 min with α-Fe crystal grains of 19–30 nm in diameter. A minimum coercivity value was observed for two of the most Cu-rich alloys: Fe_85.3_Cu_0.7_B_14_ and Fe_85_Cu_1_B_14_, which is in general agreement with the Random Anisotropy Model (RAM).

According to such a model, the decrease in grains’ size (below the exchange length) can improve alloys’ magnetic properties. For Fe-based alloys, the exchange length minimum value is equal to 20–40 nm. The grain size of magnetically soft nanocrystalline alloys is 5–20 nm in diameter [11]. The grains’ size and also their distribution are responsible for magnetic properties. For example, the coercivity is increased by nonhomogeneous distribution and large grains. Therefore, many different alloying additives are used to control grains’ growth. It means that the glass forming ability, heat treatment parameters, and consequently magnetic properties are determined by alloys’ chemical composition. The influence of different chemical elements’ addition on the formation of the amorphous and nanocrystalline phase in Fe-based alloys is described by Lashgari et al. [12]. They concluded that boron and silicon improve the GFA (Glass Forming Ability); however, the addition of B above 10 at.% has a negative impact on the magnetic properties. On the other hand, the controlled Cu addition has a positive impact on the grains’ size and their uniform distribution formed during the crystallization process.

In 2007, M. Ohta and Y. Yoshizawa [5] presented magnetic results for Fe_85-x_Cu_x_B_15_ melt-spun alloys after conventional annealing. One hour of annealing at 390 °C and subsequent magnetic properties’ measurements were performed for a single sheet of ribbons and showed the relatively very high coercivity value (>200 A/m) up to the Cu = 1% content alloy and then drastically decreased for the Cu = 1.5% alloy. By comparison with crystal structure studies, the magnetic results were explained within the RAM model. The high Cu addition brought a high number density of nucleation centers of primary crystals in the as-quenched state and a reduction in the average grain size by the simultaneous grain growth by annealing.

In this work, the thermodynamic calculation approach and comprehensive studies to verify the Cu influence on the Fe_86_B_15_ binary are performed. The 20 min conventional annealing optimization and magnetic properties (magnetic saturation, coercivity, core losses, complex magnetic permeability) are investigated on the wound toroidal cores, which is especially crucial from the application point of view. The effect of Cu on the thermal stability, nanocrystallization process, and local atomic structure, by using the thermal analysis, X-ray diffractometry, microscopy observations, and Mössbauer spectrometry, is also discussed.

## 2. Materials and Methods

The amorphous alloys with nominal composition Fe_85-x_Cu_x_B_15_, x = 0,0.6,1.2,1.5 (at.%), in the form of a ribbon with a thickness of approximately 18–23 µm and width of 5–6 mm, were obtained using the melt spinning technique on a Cu wheel (650 mm diameter) in a protective atmosphere. The master alloys were prepared from pure chemical elements (Fe 3N and Cu 4N) and the FeB_18_ 2.5N alloy by use of the vacuum induction furnace SecoWarwick VIM-LAB 50-60 (SecoWarwick S.A., Swiebodzin, Poland). For magnetic measurements, the obtained amorphous ribbons were wound into toroidal cores (except the Cu = 1.5% alloy where the as-spun ribbon was amorphous but brittle) with an inner and outer diameter of about 20 mm and 30 mm, respectively. Then, the isothermal annealing process for 20 min in a vacuum furnace (1·10^−3^ mbar) at different temperatures (260–460 °C) of wound toroidal cores was performed to achieve the nanocrystalline state. For the as-spun and heat-treated ribbons, the crystal structure was studied via the X-ray diffraction (XRD) method. XRD measurements were performed at room temperature using a Rigaku MiniFlex 600 diffractometer (Rigaku, Tokyo, Japan) equipped with CuK_α_ radiation (λ = 0.1542 nm), the K_β_ Ni filter, and the D/teX ultra-high-speed silicon strip detector. The crystallization processes were monitored using the differential scanning calorimetry (DSC). DSC thermal analyses were performed with a heating rate of 10–50 °C/min using a thermal analyzer Netzsch DSC 214 Polyma (NETZSCH-Gerätebau GmbH, Selb, Germany). The transmission electron microscopy (TEM) images in the bright-field (BF) mode and selected area electron diffraction (SAED) patterns were recorded for selected annealed samples using the Tecnai G2 F20 (200 kV) electron microscope (Thermo Fisher Scientific, Waltham, MA, USA). Additionally, for the local atomic arrangement of selected crystal structure objects, the HREM (High Resolution Electron Microscopy) observations with FFT (Fast Fourier Transform) and IFFT (Inverse Fast Fourier Transform) image conversions were performed. The Remacomp C-1200 (MAGNET-PHYSIK Dr. Steingroever GmbH, Köln, Germany) magnetic measurement system was used to determine hysteresis and magnetic properties (saturation induction Bs, coercivity Hc, core power losses P_10/50_, i.e., in B = 1 T and f = 50 Hz) of the annealed samples. The measured Hc value accuracy is 0.2% of the reading value. For calculation, the specific losses P_s_, the specimen mass (*m*), or the density (*ρ*) are used. The obtained values are automatically recalculated using the measurement control software. The relations between the quantities are:

(1) $$P_{s}=\frac{P}{\rho }=\frac{P_{c}}{m}=\frac{f}{\rho }\cdot \frac{N_{1}}{N_{2}}\oint U_{i}\cdot I$$

where: P_s_ means specific loss, P—loss, P_c_—core loss, f—frequency, ρ—density, N_1_—magnetizing winding (also named as primary winding), N_2_—secondary winding, U_i_—voltage on the secondary winding, I–current on magnetizing winding. For samples annealed at characteristic temperatures, the complex magnetic permeability at room temperature, and in the frequency range f = 10^4^—10^8^ Hz, was determined with the Agilent 4294A impedance analyzer (Agilent, Santa Clara, CA, USA). ^57^Fe Mössbauer transmission spectra were recorded at room temperature using a MS96 spectrometer and a linear arrangement of a ^57^Co:Rh (25 mCi) source, an absorber, a detector, and a multichannel analyzer. The spectrometer was calibrated at room temperature with a 30 µm thick α-Fe foil. A numerical analysis of the Mössbauer spectra was performed using the MossWinn program (ver. 4.0). All measurements were performed at room temperature.

## 3. Results and Discussion

A thermodynamic calculation approach was performed to understand better the influence of Cu content in Fe_85-x_Cu_x_B_15_ on crystallization kinetics, crystal structure, and magnetic properties. The trial-and-error method can be applied to understand the influence of different chemical elements on amorphous phase formation and crystallization mechanisms for various alloying systems. Accordingly, many experiments are necessary to provide a comprehensive analysis. The thermodynamic approach was recently proposed and validated for different alloys based on the Fe, Fe-Co, and even Al to avoid this situation. Interestingly, this approach can obtain information about the formation of amorphous and quasicrystalline phases and crystallization from the liquid state, avoiding numerous experiments [13,14,15]. Three thermodynamics parameters, configurational entropy (Δ*S^config^*), Gibbs free energy of amorphous phase formation (Δ*G^amorph^*), and Gibbs free energy of mixing (Δ*G^mix^*), were calculated according to the Equations (2)–(4) (for more details and explanations, see [15]):

(2) $$∆S^{conf}=-R\overset{n}{\underset{i=1}{\sum }}c_{i}lnc_{i}$$

(3) $$∆G^{mix}=∆H^{mix}-T∆S^{conf}$$

(4) $$∆G^{amorph}=∆H^{amorph}-T∆S^{conf}$$

where: $∆H_{}^{mix}\;$ is the mixing enthalpy; *c_i_* is the concentration of *i*-th elements; *n* is the number of chemical elements in the alloy (in this study, *n* = 3); $∆H_{}^{amorph}$ is the amorphization enthalpy; *R* is the gas constant; and *T* is the average casting temperature of the alloy from a liquid state.

**Figure 1:**
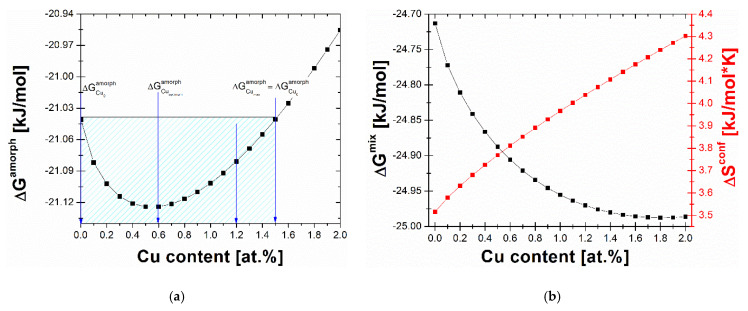
(**a**) The Gibbs free energy of amorphization (Δ*G^amoprh^*); (**b**) the Gibbs free energy of mixing (Δ*G^mix^*) and configurational entropy (Δ*S^conf^*) in the function of Cu concentration.

Figure 1 presents the results of the thermodynamic calculations. The most stable amorphous state can be obtained when the Δ*S^conf^* parameter is as high as possible, whereas Δ*G^mix^* and Δ*G^amoprh^* values are negative. From the inspection of Figure 1, it can be noted that the Cu addition causes a significant increase in Δ*S^conf^*, a decrease in Δ*G^mix^* related to the positive mixing enthalpy of the Fe-Cu system. Both tendencies contribute to the parabolic change in the Δ*G^amoprh^* with a minimum value at 0.6% of Cu. The Δ*G^amoprh^* from the Cu content dependence shape is asymmetric, and, for Cu = 0% and Cu = 1.5%, the same Δ*G^amoprh^* value is observed. It was also noticed that, obtained in this work, the minimum value Δ*G^amoprh^* = −21.12 kJ/mol at Cu = 0.6% is more negative than for the Fe_86-x_Cu_x_B_14_ system with Δ*G^amoprh^* = −20.03 kJ/mol at Cu = 0.55% studied previously [6]. Basing on obtained thermodynamic calculation results, four selected compositions were taken into consideration in the experimental part: Fe_85-x_Cu_x_B_15_, x = 0,0.6,1.2,1.5.

**Figure 2:**
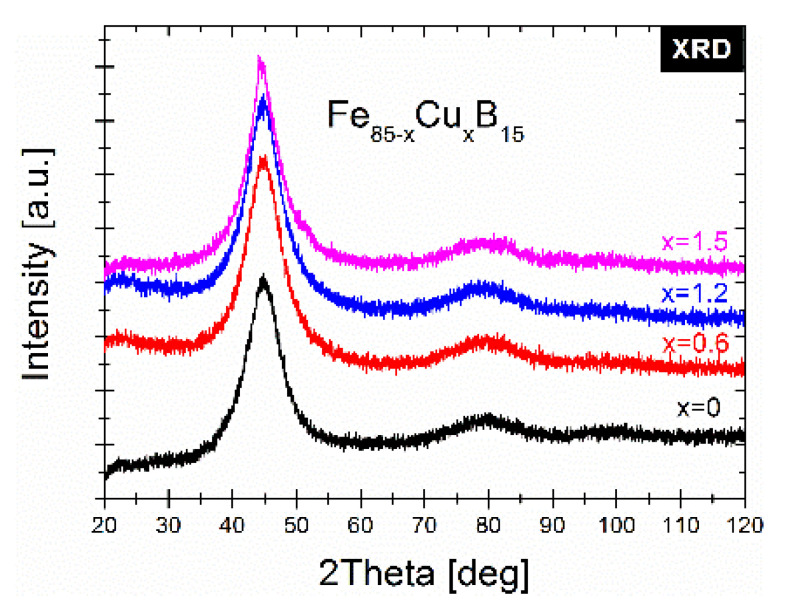
The X-ray diffraction patterns of as-spun ribbons.

**Figure 3:**
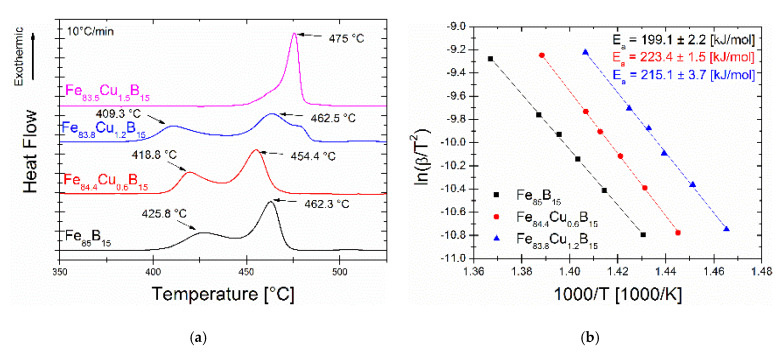
(**a**) The DSC signals of as-spun ribbons; (**b**) The Kissinger plots with the calculated activation energy of the α-Fe phase.

The XRD patterns presented in Figure 2 confirmed the amorphous state of all the studied as-spun alloys and only diffused maxima named as the amorphous halos are seen. Figure 3a depicts the DSC heat flows of the as-spun alloys. Besides the Cu = 1.5% alloy, the DSC heat flows prove the two-stage crystallization process: primary crystallization of the α-Fe phase and secondary crystallization of the borides phases. For the Cu = 1.5% alloy, the primary crystallization is unseen on the DSC curve measured with a heating rate of 10 °C/min because of the scale effect. This process is extremely diffused with a low DSC signal in the temperature range of 375–450 °C. Additionally, as it was mentioned in the experimental section, the as-spun Cu = 1.5% ribbon was very brittle and that may come from the high amount of the Cu nanoclusters as nucleation sites and the co-existence of the initial Fe clusters that tend to arrange within the α-Fe phase symmetry. Therefore, for Fe_85-x_Cu_x_B_15_ with x = 0,0.6,1.2, the kinetics of α-Fe type phase crystallization (primary crystallization peak) were studied with the use of the DSC method by performing measurements with heating rates in the range from 10 to 50 °C/min. In order to determine the average activation energies of such a non-isothermal crystallization process, the Kissinger model [16] was used. This method is based on the equation:

(5) $$ln\left(\frac{\phi }{T_{p}^{2}}\right)=ln\left(\frac{A_{0}R}{E_{a}}\right)-\frac{E_{a}}{\left(RT_{p}\right)}$$

where: $T_{p}$—temperature of the crystallization peak; ϕ—a heating rate; $E_{a}$—activation energy; R—gas constant; and $A_{0}$—pre-exponential factor. The average activation energy E_a_ of the process is determined from the slopes of linearly fitted $ln\left(\phi /T_{p}^{2}\right)$ vs. $1/T_{p}$ curves. The Kissinger plots, as well as calculated E_a_ values, are presented in Figure 3b. From the inspection of the thermal analysis, it is clearly seen that for all samples, the thermal stability (referred here as the temperature difference between the primary and secondary crystallization peaks) is relatively low of about 40–50 °C. This value is about two times smaller than what was measured previously for the Fe_86-x_Cu_x_B_14_ system [6]. This is in agreement with the general tendency of the Fe-B system where the thermal stability leads to 0 in the eutectic point for the Fe_83_B_17_ alloy. The E_a_ values fluctuate from 199 kJ/mol for the Cu = 0% alloy to 223 kJ/mol for the Cu = 0.6% alloy and 215 kJ/mol for the Cu = 1.2% alloy. Calculated E_a_ values are of the same energy range as for the Fe_86-x_Cu_x_B_14_ system [6].

**Figure 4:**
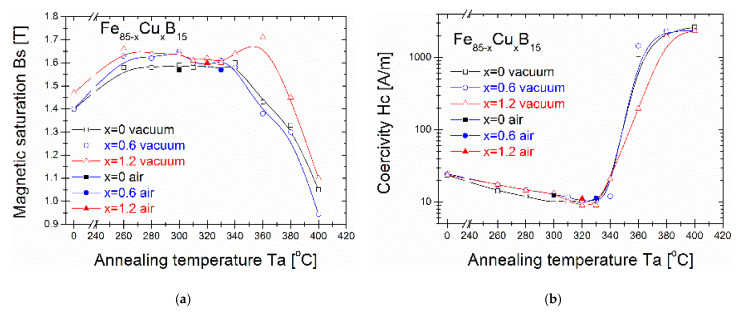
(**a**) Magnetic saturation from annealing temperature dependence; (**b**) Coercivity from annealing temperature dependence.

**Figure 5:**
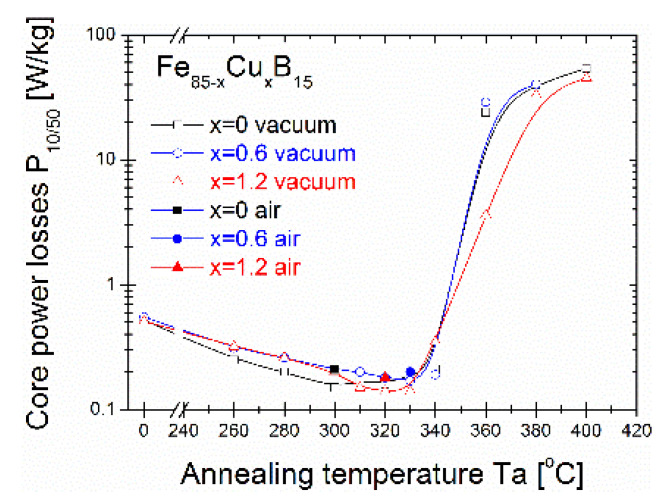
Core power losses from annealing temperature dependence.

The saturation induction Bs (Figure 4a), coercivity Hc (Figure 4b), and core power losses P_10/50_ (Figure 5) of the Fe_86-x_Cu_x_B_14_ alloys annealed for 20 min at varying annealing temperatures (Ta) are presented. It can be seen that for as-quenched alloys, saturation induction increases from 1.4 T to 1.47 T with the Cu content. Bs values of all three alloys rise substantially even at Ta = 260 °C and then slightly decrease with increasing Ta up to 330 °C. Then, from Ta = 340 °C, saturation induction drastically changes with a strong drop at 400 °C to 0.9-1T. The maximum induction saturation value reaches ~1.7T for the Cu = 1.2% alloy at Ta = 360 °C. Taking a deeper insight into Hc(Ta) and P_10/50_(Ta) (Figure 4b and Figure 5, respectively) dependences, a strong jump can be observed in both values (log scale) in the same Ta region (340–400 °C) where Bs values decrease. The global minimum of the P_10/50_ values is found at 300 °C (P_10/50_ = 0.15 W/kg) for the Fe_85_B_15_ alloy, at 330 °C (P_10/50_ = 0.17 W/kg) for the Fe_85_Cu_0.6_B_15_ alloy, and at 320 °C (P_10/50_ = 0.14 W/kg) for the Fe_83.8_Cu_1.2_B_15_ alloy. For all optimally annealed alloys, the Hc value is below 10 A/m. By increasing the annealing temperature to 380 °C, the Hc value achieves over 2000 A/m and P_10/50_ over 10 W/kg. The optimal soft magnetic properties are strongly deteriorated for Ta > 340 °C. Additionally, for optimal annealing conditions defined by Ta with a global minimum P_10/50_ value, the air-annealing process was performed as a test. Presented results of magnetic properties proved that all studied materials are sufficiently resistant to oxygen content during annealing in the temperature range 300–330 °C for 20 min. It might be a useful and alternative annealing process option applicable on an industrial scale.

**Figure 6:**
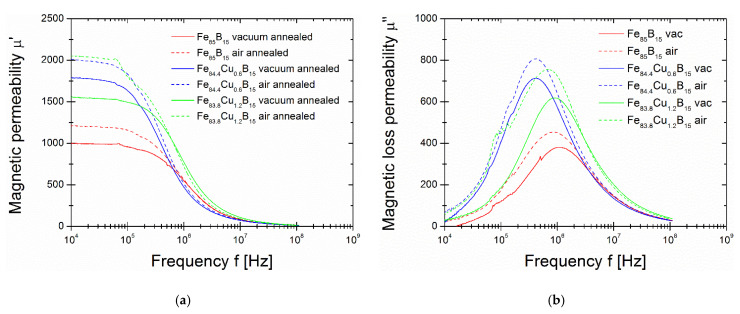
(**a**) Magnetic permeability for air- and vacuum-annealed at P_10/50_ optimum conditions of three different alloys; (**b**) Magnetic loss permeability for air- and vacuum-annealed at P_10/50_ optimum conditions of three different alloys.

Figure 6a shows real part µ’ of the complex magnetic permeability, while Figure 6b shows the µ” defined as the magnetic loss permeability of alloys at optimally annealed stages (both in vacuum and air) (a) and for annealing close to the second–local minimum of P_10/50_(Ta) dependence at 460 and 500 °C in vacuum (right panel). The vacuum-annealed has a slightly lower µ’ value than their air-annealed counterparts. The vacuum-annealed binary Fe_85_B_15_ alloy has µ’ = 1000; then, with an increasing Cu content to 0.6%, µ’ increases over 1750 and then decreases with a Cu content of 1.2% to less over 1500. This is generally in agreement with optimally annealed other Si-free Fe-based soft magnetic materials where the level of magnetic permeability µ’ is less than 10^4^ [6,7,17]. For air-annealed samples, this value is 250–500 higher than for vacuum-annealed samples. Maximum values of µ” named as the cut-off frequency are defined usually as the working frequency of materials and are in the frequency range from 4·10^5^ to 10^6^ Hz.

**Figure 7:**
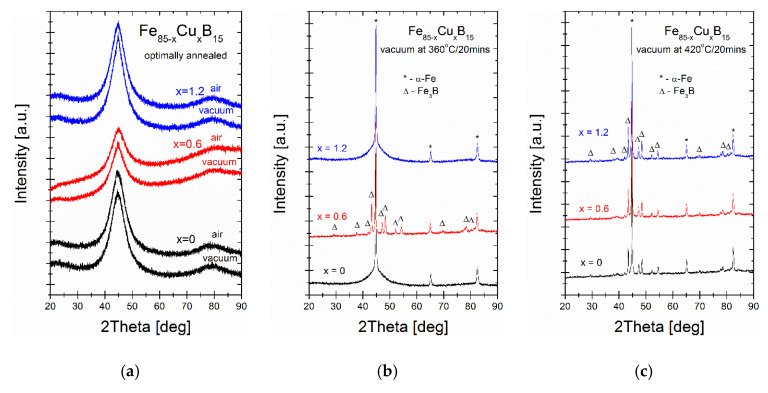
The X-ray diffraction patterns of alloys air- and vacuum-annealed at different temperatures: (**a**) annealed at optimal temperatures; (**b**) annealed at 360 °C; (**c**) annealed at 420 °C. The α-Fe and boride phase was identified and marked on patterns.

As it was mentioned in the introduction section, according to the RAM model, the magnetic properties strongly correlate with the magneto-crystalline anisotropy value of the recrystallized phase and diameters of the crystal grains. To verify the crystal structure evolution, the XRD measurements were performed at an optimally annealed state and also at two higher temperatures: 360 and 400 °C. From the inspection of XRD patterns recorded for optimal annealing temperatures (Figure 7), the fully amorphous state is confirmed. This is reflected in XRD patterns as a diffused diffraction halo. The crystal structure of vacuum-annealed alloys at 360 °C proves the presence of a well-crystallized α-Fe phase with a small contribution of the amorphous matrix for Cu = 0% and Cu = 1.2% alloys, while for the Cu = 0.6% alloy, the two-phase (α-Fe + Fe_3_B) system exists. Both phases’ peaks were identified in XRD patterns of all alloys annealed at 400 °C.

**Figure 8:**
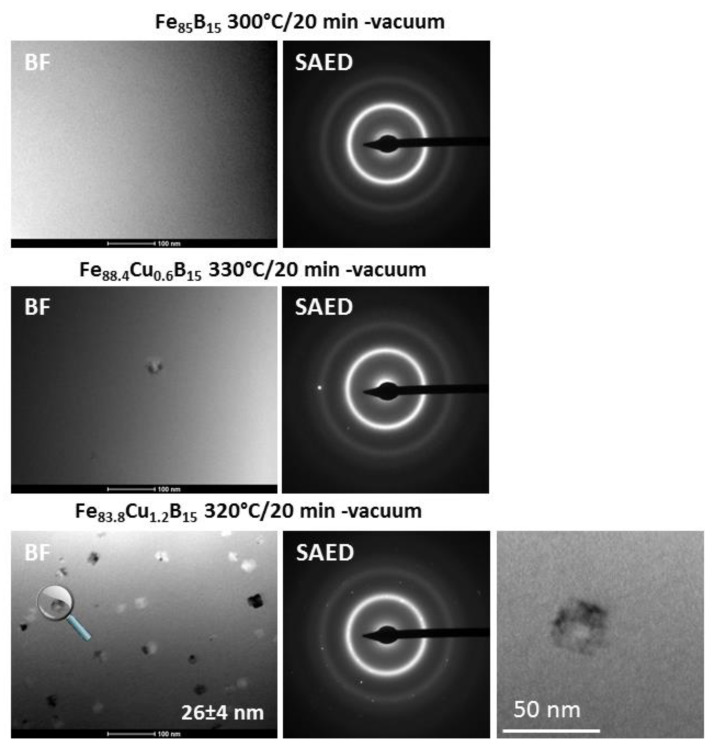
Set of BF images and SAED patterns for optimally annealed ribbons. The selected nanocrystal is magnified for the Fe_83.8_Cu_1.2_B_15_ alloy.

**Figure 9:**
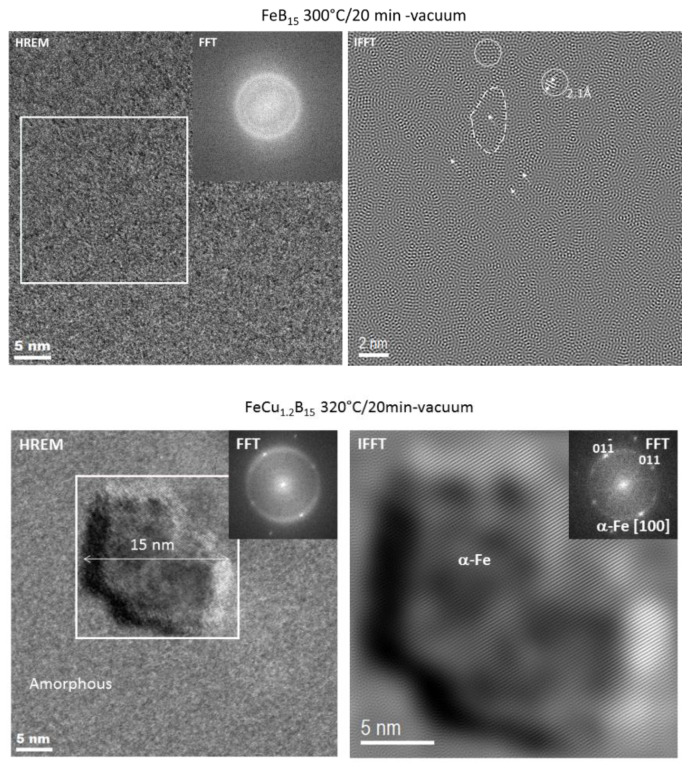
HREM and IFFT images for optimally annealed Fe_85_B_15_ and Fe_83.8_Cu_1.2_B_15_ alloys.

A more detailed crystal structure analysis focused on a local structural arrangement brought on by the TEM observations. Bright-field (BF) images and selected area electron diffraction (SAED) patterns for optimally annealed samples are shown in Figure 8. It can be seen from the BF TEM images and SAED patterns that there is a fully amorphous structure in the optimally annealed Fe_85_B_15_ alloy, whereas a low volume fraction of α-Fe nanocrystals in Fe_84.4_Cu_0.6_B_15_, and significantly more of the α-Fe nanocrystals are randomly distributed within the residual amorphous matrix. A statistical analysis and the careful crystal size determination by using the Gatan Digital Microscopy suite help to determine the average nanocrystals’ size of about 26 nm. To verify the local atomic arrangement of selected crystal structure objects, the high-resolution TEM with FFT (Fast Fourier Transform) and IFFT (Inverse Fast Fourier Transform) image conversions were performed. The deeper insight into an optimally annealed Fe_85_B_15_, shown in Figure 9, confirmed the presence of short/medium range order areas (3–4 nm) indicating the early stage of the nanocrystallization process. One can see two types of ordering: (i) nanoclusters (indicated dotted line) and (ii) an onion-like (indicated by arrows) contrast creating a specific pattern, aligned in one dominating direction. However, in some places, nanocrystals with well-defined cross-grating of crystallographic planes can be distinguished. The measured interplanar spacing of about 2.1 Å very well fits to (110)_α-Fe_ crystallographic planes. Thus, it can be stated that only the atomistic level observations helped to find the real origin of good magnetic properties of the Fe_85_B_15_ alloy. The HREM image together with FFT and IFFT of the selected nanograin of the Fe_83.8_Cu_1.2_B_15_ alloy is presented also in Figure 9. A 15 nm nanograin was identified in detail by FFT and IFFT within the symmetry of the α-Fe phase. 

Based on detailed crystal structure studies and observations, it is possible to interpret the magnetic properties at different annealing states. Identified at an optimally annealed state, the α-Fe phase is ferromagnetic, while identified at higher temperatures, the Fe_3_B phase belongs to the hard-magnetic phase [18,19]. During the annealing process, the atoms rearrange locally and form atomic short-range ordered clusters that are immersed in the amorphous matrix. The coupling of formed clusters leads to anisotropy. In this work, the optimal annealing process, defined at the least lossy materials state, was performed at different temperatures for different Cu for Fe substitution. As it was mentioned in the introduction section, Cu atoms play a crucial role as nucleation centers for α-Fe phase formation. Results presented in this work showed that for optimally annealed samples, the α-Fe phase exists in a completely different state of the nanocrystallization process. For the Cu-free alloy, the least lossy crystal structure is related with 3–4 nm short-ordered clusters; for the Cu = 0.6% alloy, only the limited value of several α-Fe nanograins are found, while for the Cu-rich alloy with Cu = 1.2%, the average diameter of nanograins is about 26 nm, and they are randomly distributed in the amorphous matrix. Coercivity values for all of these samples were at a similar level, 9.5–10 A/m. It means that there should be a proper balance between the amount and size of formed nanograins (or even atomic clusters) of differently recrystallized nanocomposite material. It has to be noticed that, for all the samples at optimally annealed states, the XRD patterns did not show any evidence (within the method accuracy) of the crystallization process. Only atomistic level observations proved the local structural arrangement. Going to the higher temperature of annealing crystal state, at 360 °C, the high number of nucleation sites in the Cu = 1.2% alloy allows for further grains’ coarsening of the α-Fe phase that is seen on the increasing value of Hc and P_10/50_ in Figure 3b and Figure 4, but also strongly enhanced the ferromagnetic exchange between the α-Fe nanocrystals, what is clearly seen on the increasing value of saturation induction in Figure 3a. For the Cu-free alloy, due to a lack of proper amounts of nucleation sites, the improper α-Fe coarsening decreases the saturation induction as well as the Fe_3_B phase co-precipitated in the limited Cu content Cu = 0.6% alloy, where calculated thermal stability was extremely low at 35 °C. For samples annealed at Ta = 400 °C, the strong deterioration of magnetic properties is related to high boride phase content with improperly formed α-Fe grains in a very narrow temperature window of thermal stability.

**Figure 10:**
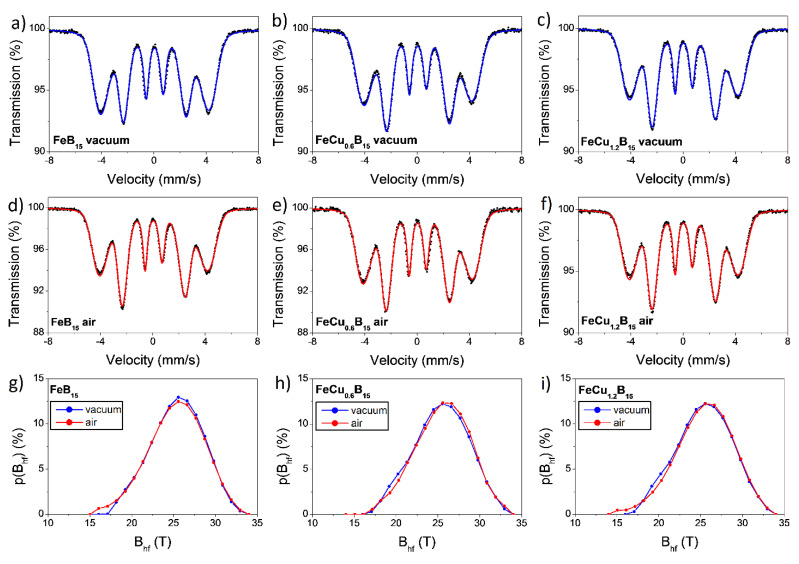
The room temperature Mössbauer spectra: (**a**) vacuum-annealed Fe_85_B_15_ alloy; (**b**) vacuum-annealed Fe_84.4_Cu_0.6_B_15_ alloy; (**c**) vacuum-annealed Fe_83.8_Cu_1.2_B_15_ alloy; (**d**) air-annealed Fe_85_B_15_ alloy; (**d**) air-annealed Fe_84.4_Cu_0.6_B_15_ alloy; (**f**) air-annealed Fe_83.8_Cu_1.2_B_15_ alloy. Corresponding hyperfine magnetic fields distributions of air- and vacuum-annealed alloys: (**g**) Fe_85_B_15_; (**h**) Fe_84.4_Cu_0.6_B_15_; (**i**) Fe_83.8_Cu_1.2_B_15_.

Mössbauer spectra measured at room temperature of the Fe_85-x_Cu_x_B_15_ with x = 0,0.6,1.2 alloys annealed under different conditions are presented in Figure 10. Obtained Mössbauer spectra are characteristic for ferromagnetic amorphous metallic alloys and exhibit overlapped and broadened sextets of absorption lines [20,21]. The average local hyperfine magnetic field as well as the position of the net magnetic moment of the samples can be determined from absorption lines (from intensities of the second and the fifth lines). The number of B atoms can be derived from parameters of hyperfine interactions at the ^57^Fe nucleus (n_B_) of the Fe atom, since, in the Fe–B system, the hyperfine magnetic field B_hf_ is a linear function of B atoms that form the nearest neighborhood (n_B_). The Mössbauer data were fitted using a hyperfine magnetic field distribution with the quadrupole splitting averaged to zero. A linear correlation between the isomer shift (IS) and the hyperfine magnetic field (B_hf_) and of the distribution components was introduced. The hyperfine parameters of fitted Mössbauer spectra are gathered in Table 1.

The average magnetic hyperfine field is a parameter to describe the average hyperfine interaction and is proportional to the spin-exchange interaction between magnetic atoms in each sample. It is noteworthy that the average hyperfine magnetic field and isomer shift remain practically unchanged for vacuum-annealed samples and air-annealed ones. Moreover, these parameters practically did not change with different Cu contents in the investigated alloys. The almost constant values B_hf_ and Is indicate that the chemical environment of the resonant nuclei is not altered, which means that changes in the short-range order are of a topological and not of a chemical origin. The range of hyperfine magnetic fields for all the spectra is between 15 T and 34 T, and obtained distributions are almost symmetrical with the highest probability of B_hf_ located at about 25.5 T, which suggests that the Fe atom has seven to zero B atoms as nearest neighbors, but the most preferential local surrounding is three boron atoms in the nearest neighborhood of the Fe atom [22].

Obtained Mössbauer spectra and corresponding hyperfine magnetic fields distributions of investigated alloys indicate an absence of components related to pure alpha-Fe. The full line width at half maximum (FWHM) is related both to the chemical and topological disorder in the crystal lattice, manifested by the existence of many inequivalent positions of iron atoms with slightly different values of hyperfine parameters. It can be observed (Table 1) that the value of this parameter decreases after air-annealing for all investigated alloys, indicating a tendency to local ordering during this heat treatment.

The sextets of Mössbauer lines deliver information on the orientation of the net magnetic moment of the studied sample via their mutual intensities ratios, which are quantified by the angle (Θ) between the direction of the gamma beam and the vector of net magnetization in a Mössbauer effect experiment. The latter can be derived from the *z* value, which represents the relative intensity of the second and the fifth spectral line with respect to the first, sixth, and the third, fourth lines (I_1_:I_2_:I_3_:I_4_:I_5_:I_6_ = 3:z:1:1:z:3) [23,24]. It can vary between 0° and 90°, which corresponds to z = 0 and z = 4, respectively. In the case of randomly oriented spins (e.g., in powder), Θ = 54.7°, the so-called magic angle, and z = 2. It should be noted that measured samples were in the form of the ribbon; hence, the effect of texture was visible on the obtained Mössbauer spectra. Determined Θ values are presented in Table 1.

## 4. Conclusions

In this work, based on the thermodynamic prediction, the comprehensive studies of the influence of Cu for Fe substitution on the crystal structure and magnetic properties of the rapidly quenched Fe_85_B_15_ alloy were performed. The research results can be summarized as follows:

1. From thermodynamic calculations, the parabolic shape dependence of the Δ*G^amoprh^* with a minimum value at 0.6% of Cu was predicted. The Δ*G^amoprh^* from the Cu content dependence shape is also asymmetric and for Cu = 0% and Cu = 1.5%; the same Δ*G^amoprh^* value is observed.
2. The as-spun Fe_83.5_Cu_1.5_B_15_ ribbon crystal structure was identified in the amorphous state (within XRD method accuracy) but also very brittle. Therefore, the obtained ribbon cannot be wound into the toroidal cores and used for a further annealing optimization process and magnetic properties’ characterization.
3. The minimum value of core power losses P_10/50_ of optimally annealed Fe_85-x_Cu_x_B_15_ x = 0,0.6,1.2 alloys comes from a completely different crystallization state of nanocomposite materials, but it strongly correlates with Cu content and thus the number of nucleation sites. The TEM observations showed that for the Cu-free alloy, the least lossy crystal structure is related with 2–3 nm short-ordered clusters; for the Cu = 0.6% alloy, only the limited value of several α-Fe nanograins are found, while for the Cu-rich alloy with Cu = 1.2%, the average diameter of nanograins is about 26 nm, and they are randomly distributed in the amorphous matrix.
4. Because of the very low thermal stability of as-spun alloys, only the high number of nucleation sites in the Cu = 1.2% alloy allows for sufficient grains’ coarsening of the α-Fe phase that strongly enhances the ferromagnetic exchange between the α-Fe nanocrystals, which is clearly seen on the increasing value of saturation induction up to 1.7T.
5. For optimal annealing conditions (20 min annealing), there are no differences in magnetic properties between the vacuum- and air-annealed materials.
6. Mössbauer spectroscopy results indicate that Cu content did not cause visible changes in hyperfine parameters of Fe_85-x_Cu_x_B_15_ with x = 0,0.6,1.2 alloys annealed under different conditions. However, obtained results show that annealing in air leads to local ordering in investigated alloys.

## Figures and Tables

**Table 1:** Mean values of the hyperfine parameters for vacuum- and air-annealed Fe_85-x_Cu_x_B_15_ with x = 0,0.6,1.2 alloys. IS—isomer shift, B_hf_—hyperfine magnetic field, FWHM—full line width at half maximum, Θ—angle between the direction of the gamma beam and the vector of net magnetization.

Hyperfine Parameters	Fe_85_B_15_		Fe_84.4_Cu_0.6_B_15_		Fe_83.8_Cu_1.2_B_15_	
	Vacuum	Air	Vacuum	Air	Vacuum	Air
IS (mm/s)	0.051	0.048	0.057	0.053	0.052	0.050
B_hf_ (T)	25.41	25.27	25.38	25.55	25.43	25.42
FWHM (mm/s)	0.47	0.41	0.47	0.43	0.44	0.42
Θ (deg)	57.9	66.5	63.9	63.9	66.5	67.8

## Data Availability

The data presented in this study are available on request from the corresponding authors.
